# Vitrification of Human Oocytes Before or After Rescue-IVM Does not Impair Maturation Kinetics but Induces Meiotic Spindle Alterations

**DOI:** 10.1007/s43032-024-01596-7

**Published:** 2024-05-21

**Authors:** Gaëlle Marteil, Aïcha Metchat, Sandra Dollet, Camille Cugnot, Laure Chaput, Bruno Pereira, Anne Sophie Gremeau, Florence Brugnon

**Affiliations:** 1 Université Clermont Auvergne, Inserm, Imagerie Moléculaire Et Stratégies Théranostiques, UMR 1240, Clermont-Ferrand, France; 2 Université de Toulouse, Toulouse, France; 3 Assistance Médicale À La Procréation, CECOS, CHU Estaing, Clermont-Ferrand, France; 4 Délégation À La Recherche Clinique Et À L’Innovation, CHU Gabriel Montpied, Clermont-Ferrand, France

**Keywords:** Human oocytes, Vitrification, In vitro maturation, Time-lapse, Meiotic spindle, Actin

## Abstract

Cryopreservation of in vitro matured oocytes is still considered as an experimental alternative to mature oocyte vitrification after ovarian stimulation. Here, we investigated whether rescue-IVM should be performed before or after vitrification. For this, 101 immature oocytes (germinal vesicle stage) from women undergoing ICSI were used. Oocytes were divided into three groups: freshly in vitro matured oocytes (IVM), freshly in vitro matured oocytes subsequently vitrified (IVM + VIT) and vitrified/warmed GV oocytes then in vitro matured (VIT + IVM). Oocyte maturation rates and kinetics were assessed using time-lapse technology. Spindle dimensions and polarity, chromosome alignment and cytoplasmic F-actin filament length and density were determined using confocal microscopy and quantitative image analyses. No differences in IVM rates (fresh IVM: 63.16% and IVM post-VIT: 59.38%, *p* = 0.72) and timings (17.73 h in fresh IVM, 17.33 h in IVM post-VIT, *p* = 0.72) were observed whether IVM is performed freshly or after vitrification. Meiotic spindles were shorter in VIT + IVM (10.47 µm vs 11.23 µm in IVM and 11.40 µm in IVM + VIT, *p* = 0.012 and *p* = 0.043) and wider in IVM + VIT (9.37 µm vs 8.12 µm in IVM and 8.16 µm VIT + IVM, *p* = 0.027 and *p* = 0.026). The length-to-width ratio was lower in vitrified groups (IVM + VIT: 1.19 and VIT + IVM: 1.26) compared to IVM (1.38), *p* = 0.013 and *p* = 0.014. No differences in multipolar spindle and chromosome misalignment occurrence and cytoplasmic F-actin filament length and density were observed between groups. Our results suggest vitrification before or after rescue-IVM does not seem to impair maturation rates and kinetics parameters but induces meiotic spindle alterations.

**Supplementary Information:**

The online version contains supplementary material available at 10.1007/s43032-024-01596-7.

## Introduction

The reference protocol for standard IVF procedures and fertility preservation is the retrieval, after ovarian stimulation, of mature oocytes, halted at the metaphase of the second meiotic division. However, ovarian stimulation may be contra-indicated for some cancer patients requiring immediate anticancer treatment such as leukaemia and for patients suffering from polycystic ovary syndrome (PCOS) at high risk of developing an ovarian hyperstimulation syndrome (OHSS) [1, 2]. In such circumstances, in vitro maturation (IVM) of immature oocytes arrested at the prophase of the first meiotic division might be an alternative approach as several births were reported in the literature, e.g. [3–8]. In the context of fertility preservation, oocyte retrieval goes hand to hand with cryopreservation. Several studies have demonstrated that the cryopreservation step can be successfully performed before or after IVM, but the most effective approach remains unclear. Most studies showed comparable high post-vitrification survival rates between immature and in vitro-matured oocytes [9–12]. Regarding oocyte competence to support maturation, results are rather conflicting with several studies showing higher maturation rates when IVM is performed before *versus* after vitrification [10, 11, 13–15] and others inversely exhibiting similar [12, 16] or even improved maturation rates for oocytes vitrified at the immature stage [9]. Despite being interesting, these studies do not give insights on the timing of meiosis progression which is considered as marker of oocyte quality in mammals [17]. Importantly, conflicting results on the developmental competences of in vitro-matured oocytes before *versus* after vitrification have been reported. While Song et al. reported lower cleavage and blastocyst rates when IVM is performed after vitrification, Molina et al. showed similar cleavage rates but higher morula rates when IVM is performed after vitrification [9, 18].

Regarding the quality of oocyte maturation, few studies have compared the meiotic spindle organisation and chromosome alignment between mature oocytes vitrified before or after IVM. Nevertheless, these two parameters are essential for sister-chromatid segregation during the second meiotic division. While the first published qualitative analysis showed no difference in the occurrence of abnormal spindle/chromosome configurations between these two groups [10], a more recent study highlighted that abnormal spindle configurations but not abnormal chromosome configurations were more frequent in oocytes vitrified after IVM compared to oocytes vitrified before IVM [12]. Regarding cytoplasmic maturation, cytoskeleton dynamics has been particularly overlooked in studies aiming at determining the most efficient oocyte stage for vitrification. The organisation of the actin cytoskeleton is of particular interest as previous studies in mouse showed that the actin network is responsible for the metaphase II spindle anchoring at the cortex [19, 20] and prevents chromosome segregation errors during meiosis [21]. While differences in cytoskeletal arrangements and functions exist between human and rodent oocytes (e.g. meiotic spindles of human oocytes lack acentriolar Microtubule-Organising Center (MTOC) in contrast to mouse oocytes [22, 23]), a recent study confirmed the importance of actin in human oocytes as the interplay between actin and microtubules at the spindle is critical for spindle bipolarity and proper chromosome alignment [24].

The aim of this study is therefore to determine whether IVM should be performed before or after vitrification. For this purpose, we performed rescue-IVM on immature oocytes collected from ICSI cycles after controlled ovarian stimulation and hCG priming, which may have different characteristics from immature oocytes collected from non-stimulated ovaries [25]. We compared the rate and kinetics of oocyte maturation of fresh in vitro-matured oocyte to those of warmed in vitro-matured oocyte using time-lapse imaging. For this study, we also assessed spindle dimensions and polarity, chromosome alignment and cytoplasmic F-actin filament length and density in fresh in vitro-matured oocyte and in in vitro-matured oocytes vitrified before or after IVM using confocal fluorescence microscopy and quantitative image analysis.

## Material and Methods

### Experimental Design and Patients

**Fig. 1:**
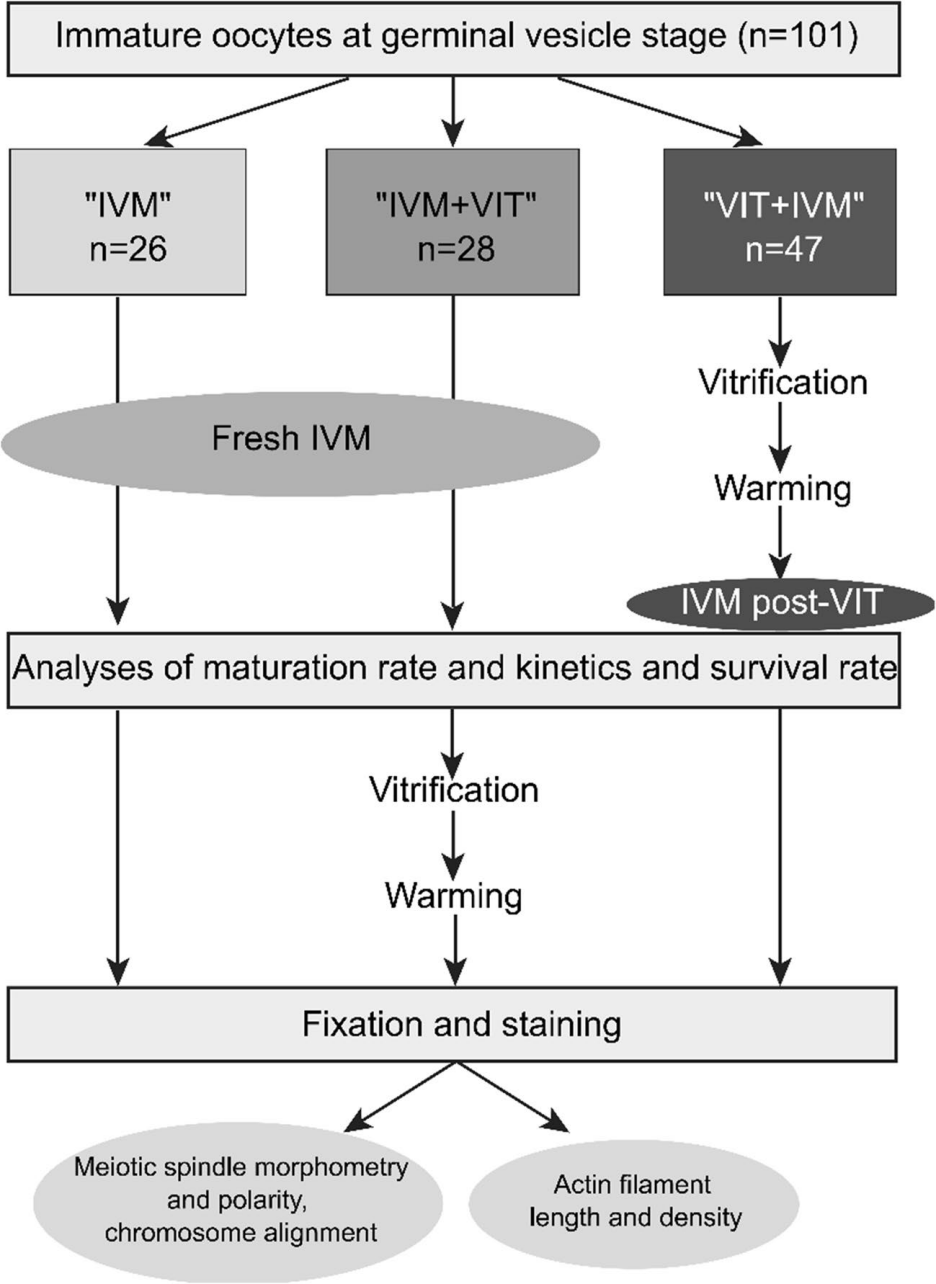
Experimental design. Immature oocytes were allocated to three groups: a control group (IVM) composed of freshly in vitro matured oocytes, a second group containing freshly in vitro matured oocytes that were subsequently vitrified (IVM + VIT) and a third group including oocytes that were vitrified at GV stage, warmed and then in vitro matured (VIT + IVM). IVM: In Vitro Maturation. VIT: Vitrification

A total of 101 immature oocytes at the germinal vesicle stage (GV) from women (< 37 years old, see Table 1 for patient characteristics by study group) undergoing intracytoplasmic sperm injection (ICSI) at the Assisted Reproductive Technology (ART) department of the University Hospital of Clermont-Ferrand, France, were used for this study. Patients with polycystic ovary syndrome, diminished ovarian reserve or severe endometriosis were excluded from this study. Immature oocytes were allocated to three groups: a control group (IVM) composed of freshly in vitro matured oocytes, a second group containing freshly in vitro matured oocytes that were subsequently vitrified (IVM + VIT) and a third group including oocytes that were vitrified at the GV stage, warmed and then in vitro matured (VIT + IVM), see Fig. 1. The minimum and maximum number of oocytes from any one patient cycle in each group were respectively 0 and 6.

**Table 1:** Patient’s characteristics and oocyte maturation and survival rates by study group

	"IVM"			"IVM + VIT"			"VIT + IVM"			*p* value	*p*_a_ value
Oocytes, n	26			28			47			-	-
Patient characteristics:	p25	Median	p75	p25	Median	p75	p25	Median	p75		
Age, years	29.92	32.00	34.66	28.75	30.92	33.10	29.33	31.83	34.50	0.14	-
Body mass index, kg/m2	20	22.5	27	20	22	24.5	23	24	28	0.03	-
Total FSH dose, IU	1232	1500	1800	1425	1500	1725	1500	1650	2250	0.97	-
Number of retrieved COCs	12	13	14	9.5	13.5	17	10	12	19	0.71	-
Maturation rate, %	63.16 (24/38)						59.38 (19/32)			0.72	0,68
Post-IVM Survival rate, %	95.00 (38/40)						82.05 (32/39)			0.09	0.08

The maturation rate corresponds to the percentage of oocytes that extruded their first polar body during the IVM step. The post-IVM survival rate corresponds to the percentage of live oocytes at the end of the IVM step. IVM and survival rates were obtained from the oocytes whose IVM was followed by time-lapse (numbers in brackets). IVM: In Vitro Maturation, VIT: Vitrification, COCs: Cumulus-oocyte complexes, FSH: Follicle-Stimulating Hormone, IU: International Unit. p25 and p75 correspond to the 25th (first quartile) and the 75 h percentile (third quartile), respectively. *Kruskal–Wallis tests were used for the comparison of patient’s characteristics and random effect models for the comparison of maturation and post-IVM survival rates without (p values) or with adjustment for the stimulation protocol (p*_*a*_* values)*

### Ethical Approval

The study protocol was approved by the Institutional Review Board of GERMETHEQUE biobank (ClinicalTrials.govID NCT03416400), site of Clermont-Ferrand. Written and informed consent was obtained for each patient to use their samples in this study (CPP 2.15.27). The Biobank has a declaration DC-2021–4820 and an authorization AC-2019–3487. The request’s number made to GERMETHEQUE is the 20170202.

### Oocyte Collection

Patients received either a long agonist or antagonist protocol, depending on the indication and their ovarian reserve. In the long protocol, the agonist (Decapeptyl® 3 mg, Ipsen Biotech, France) was started on the first day of menses, and 21 days later, after a control ultrasound, daily injections of FSH (either recombinant Gonal F®, Merck Serono, Germany, or Puregon®, Organon, Netherlands, or Bemfola® Gedeon Richter, Hungary or hMG Menopur® Ferring, Switzerland or Fertistarkit®, Ibsa, France), were started for 8 days, followed by ultrasound monitoring. The FSH dose was defined according to AMH levels. Injections continued until the trigger criteria were met (at least 3 follicles greater than 17 mm). Triggering was performed by an injection of hCG (Ovitrelle®, Merck Serono, Germany). In the antagonist protocols, pre-treatment with the contraceptive pill for 16 days was systematically performed, followed by daily FSH injections, monitoring was started at D6 and a GnRH antagonist (Cetrotide, Merck Serono, Germany) was added as soon as a follicle measured more than 14 mm to prevent premature LH surge. Triggering was performed with Ovitrelle® or Decapeptyl® 0.1 mg (two ampules), depending on the risk of developing ovarian hysperstimulation syndrome. After 36 h, the follicles were punctured vaginally under local or general anaesthesia. Cumulus cells were then enzymatically (Hyaluronidase, 80 IU/ml; Hyase, Vitrolife, Sweden) and mechanically removed. Intact GV stage oocytes were allocated to one of the three study group. An oocyte was considered intact if it exhibited a normal zona pellucida, a clear and moderately granular cytoplasm and a regular perivitelline space.

### Rescue-IVM Rate and Kinetics Measurements

**Fig. 2:**
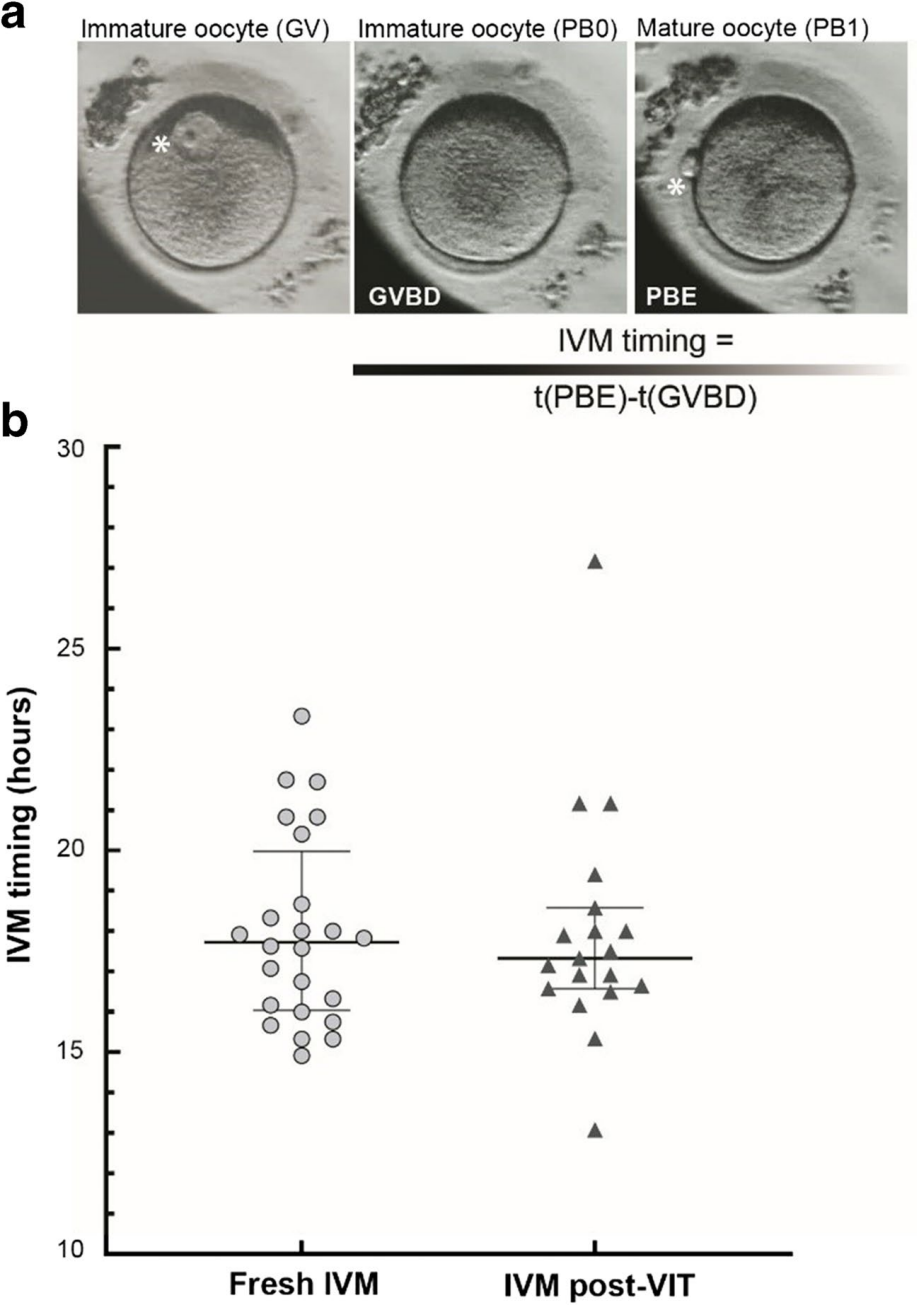
No difference in rescue-IVM timings between Fresh IVM and IVM post-VIT groups. **a** Time-lapse images of an immature oocyte (GV stage, left image) that matured in vitro (first PBE, white asterisk in the right image). IVM timing was defined as the duration between the GVBD and the first PBE. **b** Comparison of IVM timings (hours) between fresh IVM (*n* = 24, groups IVM and IVM + VIT) and IVM post-vitrification (*n* = 19, group VIT + IVM). The bar represents the median with interquartile range. *(random-effects model, p* = *0.72, p*_*a*_ = *0.58)*

GV stage oocytes were transferred to PrimoVision culture dishes (Vitrolife, Sweden) containing IVM medium (IVM System, Medicult, Origio, Denmark) supplemented with 10 mg/mL of Human Serum Albumin (Origio, Denmark) and with 0,75 IU/mL of FSH and LH (Menopur, Ferring, Switzerland). Cultures were monitored with PrimoVision time-lapse Monitoring System (Vitrolife, Sweden) and performed under 6% CO2 and 5% O2 at 37 °C for a subset of oocytes (40 for the fresh IVM group and 39 for the VIT + IVM group). The maturation status of the oocytes was checked around 26 h after putting the oocytes in the IVM medium. If oocytes had extruded their first polar body at least 2 h before the checkpoint, they were harvested and fixed for further analyses. If oocytes remained immature, the culture was extended for an additional 24 h. Images were acquired every 10 min allowing the subsequent assessment of the IVM timing which was defined as the duration between the germinal vesicle breakdown (GVBD) and the first polar body extrusion (PBE), see Fig. 2. Each movie was analysed by two examiners and the final IVM timing is the average of the two assessments. The IVM rate was defined as the number of oocytes that mature (first polar body clearly visible) during the IVM step over the total number of oocytes that were intact at the end of the IVM step. The IVM survival rate was defined as the total number of intact oocytes at the end of the IVM step over the total number of oocytes transferred in IVM medium.

The rescue-IVM of the remaining oocytes (14 in the fresh IVM group and 8 in the VIT + IVM group, so in total 22 out of 101 included oocytes) were not followed by time lapse due to space constraints in the PrimoVision time-lapse Monitoring System.

### Vitrification and Warming of Immature and Mature Oocytes

Immature and in vitro matured oocytes were vitrified with the RapidVit Omni Kit (Vitrolife, Sweden) according to the manufacturer’s recommendations. Briefly, oocytes were suspended in the equilibration medium 1 during 5 to 20 min at 37 °C and then transferred to vitrification 2 (2 to 5 min, 37 °C) and vitrification 3 (30–45 s, 37 °C) medium containing propanediol and ethylene glycol as cryoprotectants and sucrose. Oocytes were subsequently loaded in Rapid-I straws (Vitrolife, Sweden) and stored in liquid nitrogen until analysis. Oocytes were warmed using the RapidWarm Omni kit (Vitrolife, Sweden) according to the manufacturer’s recommendations. Briefly, oocytes are placed in Warm 1™ Omni medium for 1 min, then in Warm 2 ™ Omni medium for 3 min, subsequently in Warm 3 ™ Omni medium for 5 min and finally in Warm 4 ™ Omni medium for 5 to 10 min; all steps are performed at 37 °C. While Warm 1™ Omni, Warm 2™ Omni and Warm 3™ Omni contain sucrose, Warm 4™ Omni does not. Afterwards, the warmed oocytes were incubated in G-TL medium (Vitrolife, Sweden) under 6% CO2 and 5% O2 at 37 °C conditions for 2 h. After this incubation, oocytes were considered alive if they exhibited no dark/degenerated or contracted ooplasm and no alteration of zona pellucida (observation under inverted microscope at × 40 magnification). Intact mature oocytes were then directly fixed for spindle and actin analyses (see below for experimental details) and intact immature oocytes at GV stage underwent an IVM step (see above for experimental details).

### Immunofluorescence Staining and Confocal Microscopy

All reagents were purchased from Sigma (USA), unless otherwise specified.

Oocytes were fixed for 30 min at 37 °C in 100 mM HEPES, 50 mM EGTA (pH7; titrated with KOH), 10 mM MgSO4, 2% formaldehyde (methanol free) and 0.2% Triton X-100. After permeabilization in Phosphate-Buffered Saline + 1% Triton X-100 (PBT) overnight at 4 °C, oocytes were stained with anti-α-Tubulin antibodies (T6074, 1/100 dilution in PBT with 3% bovine serum albumin (BSA)). After washes in PBT + 3% BSA, oocytes were incubated with anti-mouse-Cy3 secondary antibodies (Jackson Immunoresearch, UK) and with Alexa-Fluor-488-Phalloidin (A12379, Molecular Probes, USA) diluted at 1/500 and 1/100 in PBT + 3% BSA, respectively. After washes in PBS, oocytes were finally transferred to glass bottom dishes (WillCo-Dish, Willco Wells, UK) and stained with Topro 3 (DNA staining, excitation/emission 642/661 nm, Molecular Probes, USA) for imaging. Images were acquired with a customized Leica TCS SP5 confocal microscope using the HCX PL APO CS 63.0 X 1.2 water immersion objective lens, with laser Argon, laser DPSS 561, and laser HeNe 633 illuminations.

For the Actin filament network (Phalloïdin staining), images were taken as Z-stacks (19 z-confocal sections every 0.25 μm) to visualize the entire length of actin filaments. The filament length between two nodes or between a node and the end of the filament in the cytoplasm was measured using Fiji. Please note that the cytoplasmic background was subtracted using “background subtraction” in Fiji software to enhance the signal of filament staining. For the cytoplasmic F-Actin network density, after background subtraction and segmentation of actin filament, the cytoplasmic F-actin density was analysed using the Skeleton 2D plugin’ of Fiji software. The density of actin was calculated using the following formula: d = (total length of actin filament/image area) × 100. Five different sections per oocyte were analysed.

**Fig. 3:**
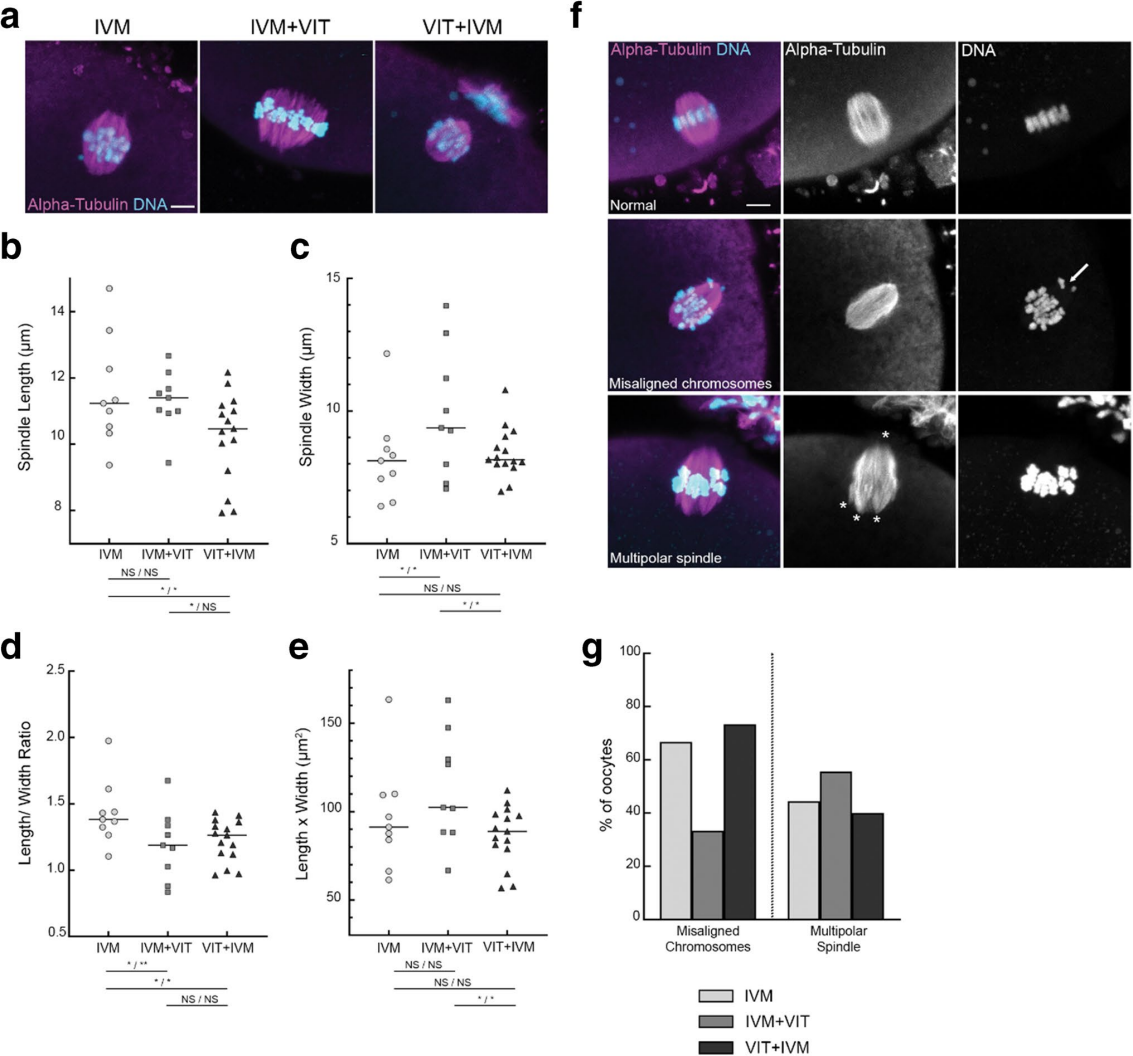
Vitrification induces meiotic spindle alterations. Meiotic spindle and chromosomes were visualized using anti α-Tubulin antibodies (Magenta) and Topro 3 (Cyan) staining, respectively. **a** Meiotic spindles of oocytes from each study group. Scale bar 5 µm. **b**-**c** Spindle width and length in the IVM (*n* = 9), IVM + VIT (*n* = 9) and VIT + IVM (*n* = 15) groups. Bars represent medians. *(random-effects model, p and p*_*a*_ values are at the left and the right of the slash, respectively; NS not significant, * p/p_*a*_ < *0.05)*. **d**-**e** Length/width ratios and length x width. Bars represent medians. *(random-effects model, p and p*_*a*_ values are at the left and the right of the slash, respectively; NS not significant, * p/p_*a*_ < 0.05, ** p/p_*a*_ < *0.01).* **f** Images of a normal spindle (bipolar, well-aligned chromosomes – upper panel), a spindle with misaligned chromosomes (middle panel, white arrow) and of a multipolar spindle (lower panel, white asterisks). Scale bar 5 µm. **g** Percentage of multipolar spindles and misaligned chromosomes in oocytes from the IVM (*n* = 9), IVM + VIT (*n* = 9) and VIT + IVM (*n* = 15). *(Fisher’s exact test, p* = *0.161 and p*_*a*_ = *0.198 for misaligned chromosomes and p* = *0.836 and p*_*a*_ = *0.473 for multipolar spindle)*

For the spindle (α-tubulin staining), a subvolume of the oocyte covering the entire spindle was imaged, with 0.5 μm z-confocal section resolution and 512 × 512 pixel in xy dimension. Spindle length and width were measured in 3D using Imaris Software. Spindle length was defined as the distance between the poles and the spindle width is the thickness of the microtubules perpendicular to the spindle axis, at the metaphase equator (see Fig S1). Three measurements were performed for each parameter and the final values are the average of the three measurements. Meiotic spindle polarity and chromosome alignment were assessed in 3D using Imaris Software. A meiotic spindle was considered as multipolar when more than two poles were clearly visible, more precisely when there were more than two focus points of microtubules (see Figs. S2 and  3f). Chromosome were considered misaligned when they were not correctly aligned or totally outside of the metaphase equator (Fig. 3f).

For all these analyses (spindle morphometry and polarity, chromosome alignment and actin filament length and density), only the oocytes that survived the IVM and/or cryopreservation steps were included.

### Statistical Analyses

Continuous data were expressed according to their statistical distribution with median and interquartile range (IQR p25-p75). The assumption of normality was analysed using the Shapiro–Wilk test. The comparisons of continuous variables between groups (IVM, IVM + VIT and VIT + IVM) for patient’s characteristics were performed using the Kruskal–Wallis test as the assumptions required to apply analysis of variance were not met. The homoscedasticity was analysed using the Bartlett test. When appropriate (omnibus p-value less than 0.05), Dunn’s post-hoc test for multiple two by two comparisons was applied. For the comparison of presence of multipolar spindles and misaligned chromosomes between groups, Fisher’s exact test was performed. Concerning correlated data (e.g. actin filament length and density, IVM timings, spindle length, width and size, length/width ratio), random-effects models were used to account for the between and within patient variability caused by several measures for the same subject. Group was considered as fixed effect whereas subject was considered as random effect. Multivariate analyses were conducted using random-effects models with the type of stimulation protocol as adjustment covariate to take into account its possible influence. The p-values associated to these multivariate analyses are depicted as “p_a_” for adjusted p-value throughout the manuscript. The statistical analyses were performed using Stata software version 15 (StataCorp, College Station, US). Statistical tests were two-sided with the type-I error set at 5% corrected, when necessary, as aforementioned.

## Results

### Patients, Samples and Experimental Design

In this study, 101 immature oocytes at GV stage from patients undergoing ICSI were analysed. To determine whether rescue-IVM should be performed before or after vitrification, immature oocytes were divided into three groups: a control group (IVM, *n* = 26) composed of freshly in vitro matured oocytes, a second group containing freshly in vitro matured oocytes that were subsequently vitrified (IVM + VIT, *n* = 28) and a third group including oocytes that were vitrified at GV stage, warmed and then in vitro matured (VIT + IVM, *n* = 47), (Fig. 1). There was no significant difference in the median age (IVM: 32.00 years, IVM + VIT: 30.92 years and VIT + IVM: 31.83 years, *p* = 0.14) and in the median total FSH dose received (IVM: 1500 IU, IVM + VIT: 1500 IU and VIT + IVM: 1650 IU, *p* = 0.97) between the different patient groups (Table 1). A slight difference in the median body mass index was observed between the three groups (IVM: 22.5 kg/m^2^, IVM + VIT: 22 kg/m^2^ and VIT + IVM: 24 kg/m^2^, *p* = 0.03). While high BMI can affect oocyte quality and result in poor oocyte retrieval, there was no significant difference in the median number of retrieved cumulus oocyte complexes (COCs) between groups (IVM: 13, IVM + VIT: 13.5 and VIT + IVM: 12, *p* = 0.71), reinforcing the comparability of our study groups. While patients received either a long agonist or an antagonist protocol, we did not observe any major impact of the type of stimulation protocol on the experimental results of this study. Indeed, the p values associated (p_a_) to the multivariate analyses after adjusting for the type of stimulation protocol (long agonist/antagonist protocol) are very similar to the regular p values.

### Oocyte Survival, IVM Rate and Kinetics

To infer the impact of vitrification on the rate and kinetics of rescue-IVM, we compare these parameters between fresh *in-vitro* matured oocytes (fresh IVM: compilation of IVM and IVM + VIT groups) and oocytes for which IVM was performed post-vitrification (VIT + IVM group), (Fig. 1). Regarding the post-IVM survival rate (Table 1), it tends to be higher in fresh *in-vitro* matured oocytes (fresh IVM) than in vitrified/warmed oocytes (95.00% versus 82.05%, *p* = 0.09). Moreover, we did not observe any difference for IVM rates between fresh IVM (63.16%) and IVM post-VIT (59.38%), *p* = 0.72. The median IVM timing measured by PrimoVision time-lapse (Fig. 2), defined as the duration between GVBD and first PBE, was 17.73 h for fresh IVM (IQR 16.09–19.54) and 17.33 h for IVM post-VIT (IQR 16.58–18.58). Statistical analyses show no significant difference between these median IVM timings (*p* = 0.72). Altogether, our results suggest that performing a vitrification/warming step before rescue-IVM does not impair maturation rates and kinetic parameters.

### Meiotic Spindle Morphometric Parameters and Polarity, Chromosome Alignment

Our results showed no difference in IVM timings and rates whether IVM is performed before or after vitrification. While these parameters give insights on the quality of oocyte nuclear maturation, we decided to investigate further this process by quantitatively assessing spindle morphometric parameters and polarity as well as chromosome alignment using confocal fluorescence microscopy (Fig. 3) as these parameters are essential for faithful sister-chromatid segregation during the second meiotic division. The metaphase-II meiotic spindle morphometric parameters were assessed directly after IVM for IVM and VIT + IVM groups and post-vitrification/warming for the IVM + VIT group, Fig. 1. Meiotic spindles (Fig. 3b) were shorter in VIT + IVM (median length: 10.47 µm) compared with IVM and IVM + VIT (median length: 11.23 µm, *p* = 0.012 and 11.40 µm, *p* = 0.043 respectively). Moreover, meiotic spindles were statistically wider in IVM + VIT (median width: 9.37 µm) compared to IVM and VIT + IVM (median width: 8.12 µm, *p* = 0.027 and 8.16 µm, *p* = 0.026 respectively, Fig. 3c). We then calculated the length-to-width ratio which describes the shape of a spindle. The median length-to-width ratio was statistically lower in both vitrified groups (IVM + VIT: 1.19 and VIT + IVM: 1.26) compared to IVM (1.38), *p* = 0.013 for IVM + VIT vs IVM and *p* = 0.014 for VIT + IVM vs IVM (Fig. 3d). Importantly, these results suggest that regardless of whether vitrification is performed before or after IVM, it alters spindle shape. As spindle size seems to be relevant for oocyte quality and clinical outcomes [26], we estimated it for each oocyte by multiplying the length times the width (Fig. 3e). Regarding this parameter, while none of the vitrified groups (median length x width of 102.41 µm^2^ for IVM + VIT and 88.81 µm^2^ for VIT + IVM) showed statistical difference to IVM (median length x width 91.25 µm^2^), a statistical difference is observed between the two vitrified groups (*p* = 0.015).

To evaluate spindle and chromosome configurations, we performed 3D reconstruction of spindles and chromosomes using IMARIS software. While “normal” spindles exhibit two poles and well-aligned chromosomes (Fig. 3f, upper panel), spindles with misaligned chromosomes and/or multiple poles were considered defective (Fig. 3f middle and lower panels). The probability to encounter a multipolar spindle in an oocyte is relatively high in IVM as well as in both vitrified groups (44.4% in IVM, 55.6% in IVM + VIT and 40.0% in VIT + IVM). A similar trend is observed for the incidence of chromosome misalignment in oocytes, although lower in IVM + VIT (66.7% in IVM, 33.3% in IVM + VIT and 73.3% in VIT + IVM). According to our analysis, there was no statistically significant difference in the percentage of oocytes with misaligned chromosomes and in the percentage of oocytes with multipolar spindles between the three study groups (*p* = 0.161 and *p* = 0.836, respectively, Fig. 3g).

### Actin Network

**Fig. 4:**
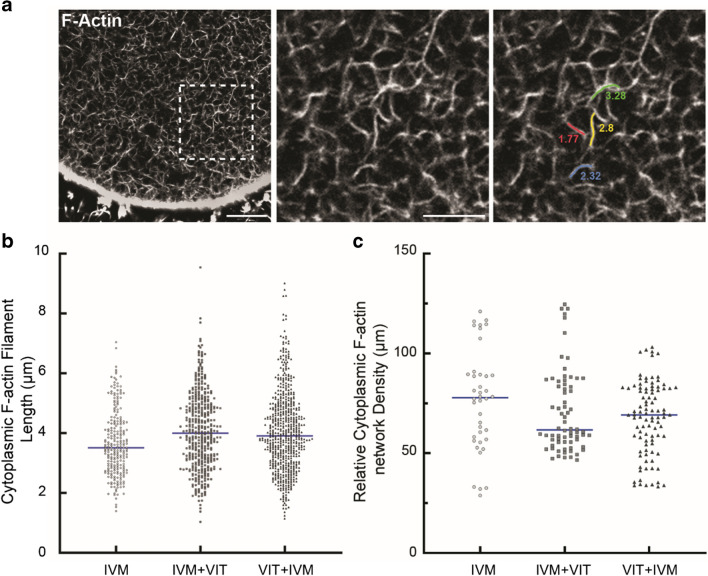
No alteration of cytoplasmic F-actin filament length and density upon vitrification. F-actin was labelled with Alexa-Fluor-488-Phalloidin. **a** Actin filaments in an oocyte from the IVM group. Scale bar 10 µm and 5 µm for the inset. **b** Actin filament length in the IVM (*n* = 240 filaments/8 oocytes), IVM + VIT (*n* = 420 filaments/14 oocytes) and VIT + IVM (*n* = 600 filaments/20 oocytes) groups. 30 actin filaments were measured per oocyte. Blue bars represent medians *(random-effects model, p* = *0.13 and p*_*a*_ = *0.16).* **c** Relative cytoplasmic F-actin network density in the IVM (*n* = 40 sections/8 oocytes), IVM + VIT (*n* = 70 sections/14 oocytes) and VIT + IVM (*n* = 95 sections/19 oocytes) groups. Five sections were analysed per oocyte. Blue bars represent medians *(random-effects model, p* = *0.50 and p*_*a*_ = *0.47)*

Oocyte competence to fertilization and early embryo development relies not only on nuclear maturation but also on cytoplasmic maturation. This process encompasses several cellular events, such as organelle redistribution, cytoskeleton dynamics and molecular maturation. Among them, we chose to analyse the actin network in this study as it has been shown to be responsible for spindle anchoring at the cortex [19, 20] and to be important for spindle bipolarity and proper chromosome alignment, therefore preventing chromosome segregation errors during meiosis [21, 24]. The actin cytoskeleton staining of the oocytes using Phalloidin revealed a dense actin network composed of small cytoplasmic filaments within the ooplasm and a thick actin layer at the cortex (Fig. 4a). No significant difference in the median cytoplasmic F-actin filament length within the ooplasm is observed between groups (3.51 µm in IVM, 4.00 µm in IVM + VIT and 3.91 µm in VIT + IVM, *p* = 0.129), Fig. 4b). We also did not observe any significant difference regarding cytoplasmic F-actin network median density between the three groups (77.83 µm in IVM, 61.70 µm in IVM + VIT and 69.18 µm in VIT + IVM, *p* = 0.50), Fig. 4c. Our results show that cytoplasmic F-actin filament length and density are not altered upon vitrification, whether this step is performed before or after rescue-IVM.

## Discussion

Our study is the first to investigate whether rescue-IVM should be performed before or after vitrification using multiparametric measurements in human oocytes. Using time-lapse imaging, we demonstrated that performing a vitrification/warming step before IVM does not impair oocyte maturation rate and IVM timing. Importantly, our high-resolution fluorescence imaging and quantitative image analyses of microtubule network of mature oocytes show that spindle shape is altered upon vitrification whether vitrification is performed before or after IVM.

In this study, IVM rates are close to those observed in studies with similar inclusion criteria as ours [9, 11, 15, 16, 27, 28] and, more importantly, no difference in IVM rates was observed between fresh and vitrified-warmed oocytes. These results are in accordance with previous studies [12, 16], therefore showing that the vitrification/warming step does not impair the oocyte ability to undergo subsequent nuclear maturation. However, fresh in vitro matured oocytes seem to survive better the IVM step than vitrified/warmed oocytes, suggesting that the vitrification step might impact the oocyte capacity to endure the IVM process. Numerous studies have indeed highlighted higher maturation rates when IVM is performed before *versus* after vitrification/warming [10, 11, 13–15], suggesting that the vitrification/warming step might interfere with the maturation progression, probably due high concentrations of penetrating cryoprotectants [29].

Our study provides the first assessment of IVM kinetics using time-lapse microscopy in vitrified/warmed human oocytes. This monitoring system allowed us to accurately follow important events of oocyte maturation such as GVBD and PBE. We could determine that IVM timings are similar between fresh and vitrified/warmed oocytes (≈ 18 h). These timings are close to those observed in human oocytes expressing fluorescent proteins allowing the visualisation of spindles and chromosomes (≈ 20.2h) [22]. So, vitrification can be safely performed either before or after rescue-IVM. Aberrant IVM timings could have reflected perturbations of intracellular pathways such as the spindle assembly checkpoint and the Anaphase Promoting Complex (APC) activity, which could trigger aneuploidy, as demonstrated in mouse [30, 31]. The future use of time-lapse in ART laboratories to monitor IVM may provide novel non-invasive criteria (e.g., GVBD, PBE and IVM timings) to evaluate the quality of in vitro matured oocytes.

In the context of fertility preservation, IVM of oocytes goes along with cryopreservation therefore the most efficient oocyte stage for cryopreservation should be defined. To address this, we evaluated metaphase II spindle size as a recent study suggested that this is a good indicator of oocyte quality in humans, having a predictive value of clinical pregnancy rate [26]. We observed no difference in spindle length x width between the three study groups, suggesting that vitrification can be performed either before or after IVM. However, Tomari et al*.* showed that oocytes with a spindle size comprised between 90 and 120 µm^2^ display higher fertilisation and clinical pregnancy rates than smaller (< 90 µm^2^) and larger spindles (> 120µm^2^) [26]. In our study, while the median spindle length x width of IVM and IVM + VIT oocytes fall in the 90–120 µm^2^ category, it is 88.81 µm^2^ in VIT + IVM oocytes, therefore suggesting that vitrification prior IVM might alter oocyte quality. Spindle length was statistically lower in the VIT + IVM group compared to the IVM and the IVM + VIT groups and spindle width was higher in the IVM + VIT group compared to the IVM and the VIT + IVM groups. Interestingly, while the impact of meiotic spindle width on human oocyte quality is not known, spindle length seems to have a positive predictive value in term of blastocyst formation, implantation and pregnancy rates [32, 33] and to be related to oocyte competence to fertilization in women with advanced maternal age [34]. In view of the literature, we can hypothesize that fertilization and development potentials of oocytes belonging to the VIT + IVM group would be lower than oocytes from IVM and IVM + VIT groups, suggesting that IVM should be performed before vitrification. Taken together, our results suggest that the vitrification step induces meiotic spindle alterations. However, supplementary studies are required to determine if these alterations are inherent to the chosen vitrification protocol (Vitrolife in our study and not the internationally standardized vitrification method [35]) or to the vitrification step itself.

We then evaluated spindle bipolarity and chromosome alignment at the metaphase plate. In line with the study of Kasapi et al. [10], our data show similar percentages of multipolar spindles and misaligned chromosome between the three study groups, therefore supporting once more that vitrification can be performed either before or after IVM. Regarding the effect of vitrification itself on spindle/chromosome configuration, our data rather support studies showing no adverse effect [36], than studies suggesting a deleterious effect [10, 16]. These discrepancies might originate from the temperature at which the vitrification and warming steps were conducted, 37 °C in our study and mostly room temperature in others. We favoured physiological temperature as it will help to maintain spindle integrity especially during the warming step as human meiotic spindles are highly sensitive to temperature fluctuations [37, 38]. In addition, the duration of the culture after PBE might account for the observed discrepancies since Yu et al. observed a higher rate of normal spindles in oocytes that were cultured from 2- to 5-h after PBE compared to 0–1 h and 8–9 h [39]. Lastly, we evaluated the actin network as it was recently shown to be essential for proper bipolar spindle assembly and chromosome segregation in human oocytes [24]. Here, we confirmed that actin is characterised by very dense cytoplasmic filaments, with long filament surrounding and penetrating the spindle and by an enrichment at the cortex [24]. We showed that cytoplasmic F-actin filament length and density are not altered upon vitrification, whether it is performed before or after rescue-IVM. To our knowledge, our study is the first one analysing actin cytoskeleton in the context of oocyte rescue-IVM and cryopreservation. Previous studies in mammals demonstrated that exposition to cryoprotectants perturbs the cortical actin of ovine oocytes and vitrification-warming compromises the actin cytoskeleton of cumulus-enclosed immature rat oocytes [11, 40, 41]. To conclude, further large-scale studies focusing on spindle morphometry, chromosome organisation and both cytoplasmic and cortical actin are still required to highlight the most efficient oocyte stage for cryopreservation.

Taken together, our results suggest that performing a vitrification step before or after rescue-IVM of human oocytes does not seem to affect maturation kinetics and cytoplasmic F-actin filaments length and density but induces meiotic spindle alterations. In this study, we used oocytes at GV stage from patients without polycystic ovary syndrome, diminished ovarian reserve or severe endometriosis. While these restrictive inclusion criteria minimize variability and confounding factors, they led to small sample size and limit the relevance of our findings. In addition, oocytes from each patient were not always dispersed across the different study groups. Consequently, larger scale studies with stricter oocyte distribution criteria are now further required to ascertain and generalize our findings. In addition, in our study, mostly invasive methods were used to study oocyte quality, preventing us from inferring fertilization and developmental competences of oocytes. Yet, these parameters are of utmost importance, especially since the occurrence of chromosome misalignments and multipolar spindles is high in our study. While part of these defects might be corrected before fertilization as we only analysed single time point images, some may remain and could lead to abnormal sister chromatid separations with subsequent aneuploid embryo formation. Several study reported that performing a vitrification step before IVM compared to after IVM has no impact on fertilization rate [9, 18, 42]. Regarding cleavage, blastocyst and high-quality embryo rates, data from the literature are rather conflicting. While Song et al. reported lower cleavage and blastocyst rates when IVM is performed after vitrification, Molina et al. showed similar cleavage rates but higher morula rates when IVM is performed after vitrification [9, 18]. Alternatively, other studies indicated that performing IVM after vitrification has no impact on fertilization and cleavage rates compared to fresh oocytes [43]. Altogether, the discrepancies observed in the literature, once again, suggest that protocols used both for IVM and vitrification/warming may have a significant impact on fertilization and developmental competences of oocytes, and consequently on the fertility preservation efficiency.

Investigating the quality of oocyte cytoplasmic maturation, notably organelle redistribution and molecular maturation using global approaches, is now critical to achieve a better picture of the impact of vitrification on oocytes. This is of particular interest since IVM of immature oocytes combined with vitrification is still currently considered as an experimental procedure. Optimizing this procedure will be a great asset especially in the context of fertility preservation or during standard IVF when immature oocytes are retrieved.

## Supplementary Information

Below is the link to the electronic supplementary material.

Supplementary file1 (DOCX 769 KB)

## Data Availability

The data that support the findings of this study are available within the article or available from the authors upon request.
