# Supplementary material for: Vitrification of Human Oocytes Before or After Rescue-IVM Does not Impair Maturation Kinetics but Induces Meiotic Spindle Alterations
Source: Reprod Sci. 2024 May 21;31(9):2625–36. doi: 10.1007/s43032-024-01596-7 (PMC11392996; doi:10.1007/s43032-024-01596-7)
Supplement: Supplementary file 1 — Supplementary file1 (DOCX 769 KB) [file 43032_2024_1596_MOESM1_ESM.docx]

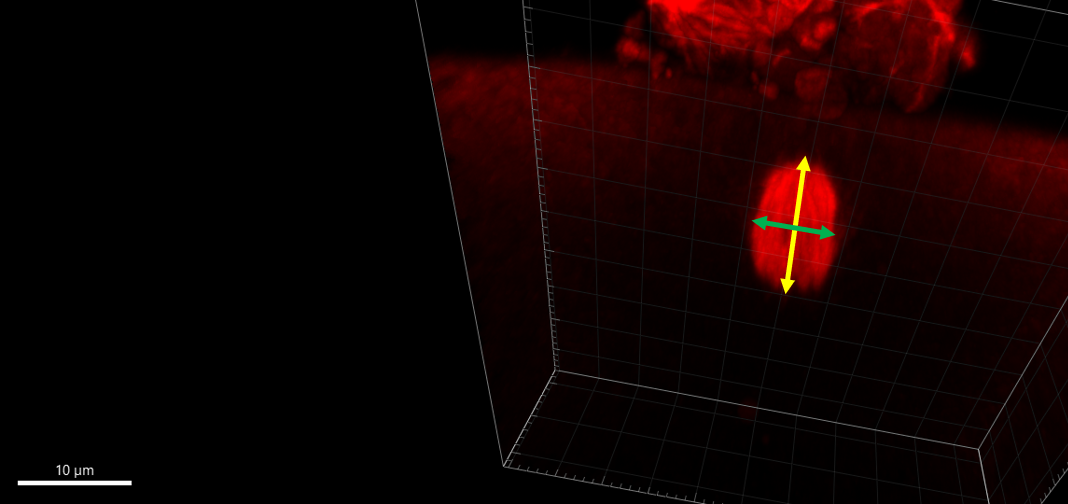


**Fig. S1:** Spindle dimension assessment. Spindle length was defined as the distance between the poles and the spindle width is the thickness of the microtubules perpendicular to the spindle axis, at the metaphase equator. Three measurements for each parameter were performed in 3D using Imaris Software and the final values are the average of the three measurements.


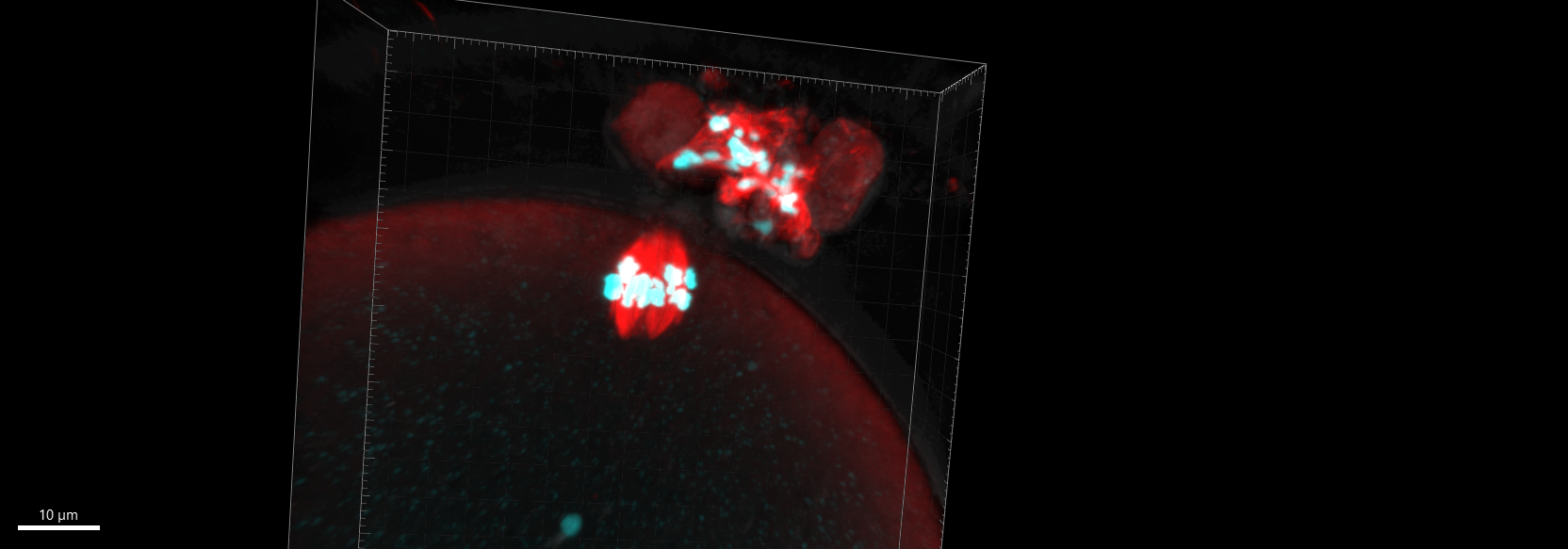


*

*

*

**Fig. S2:** Example of a multipolar meiotic spindle. A meiotic spindle was considered as multipolar when more than two poles are clearly visible, more precisely when there are more than two focus points of microtubules (see white asterisk). The analysis was done in 3D using Imaris software.
